# Feature selection and prediction with a Markov blanket structure learning algorithm

**DOI:** 10.1186/1471-2105-14-S17-A3

**Published:** 2013-10-22

**Authors:** Yuan Tan, Zhifa Liu

**Affiliations:** 1 Department of Statistics, Mississippi State University, Starkville, MS 39759, USA; 2 Department of Biostatistics, St. Jude Children’s Research Hospital, Memphis, TN 38105, USA

## Background

Classification and prediction are common tasks in machine learning. For example, many studies have attempted to predict gene expression given information, such as DNA sequence, expression of other genes or epigenetic modifications. Many existing methods, such as neural networks and support vector machines, have been used to make these predictions. Unfortunately, these black box techniques offer little insight into the reasoning behind the predictions. In many cases, relatively few attributes contribute to the classification accuracy. Bayesian networks explicitly encode the relationships among attributes to make predictions. In a Bayesian network, the Markov blanket (MB) of the class variable gives all of the information necessary to predict its value. In this work, we propose an algorithm to learn only the MB of the class variable; other attributes are removed. Therefore, our algorithm combines classification and feature selection. Results on benchmark machine learning datasets indicate that our feature selection technique usually reduces the size of the dataset more than 80% on some datasets. Accuracy results suggest that the classification ability of our algorithm is competitive with existing state of the art techniques.

## Materials and methods

In a classification problem, we are given a dataset consisting of a set of attributes **A** and a class variable *C*. Furthermore, the dataset is split into a training set *D_tr_* and a testing set *D_te_*. The goal is to learn a classifier from *D_tr_* that correctly predicts *C* in *D_te_*. In this study, we compared the performance of our Markov Blanket structure with other classical classifiers such as C4.5 [1] , optimal Bayesian network [2], and Tree Augmented Naïve Bayes Network [3] and Markov Blank Hill Climbing [4]. Here is a general introduction for those classifiers.

## Markov blanket feature selection algorithm

**Figure 1:**
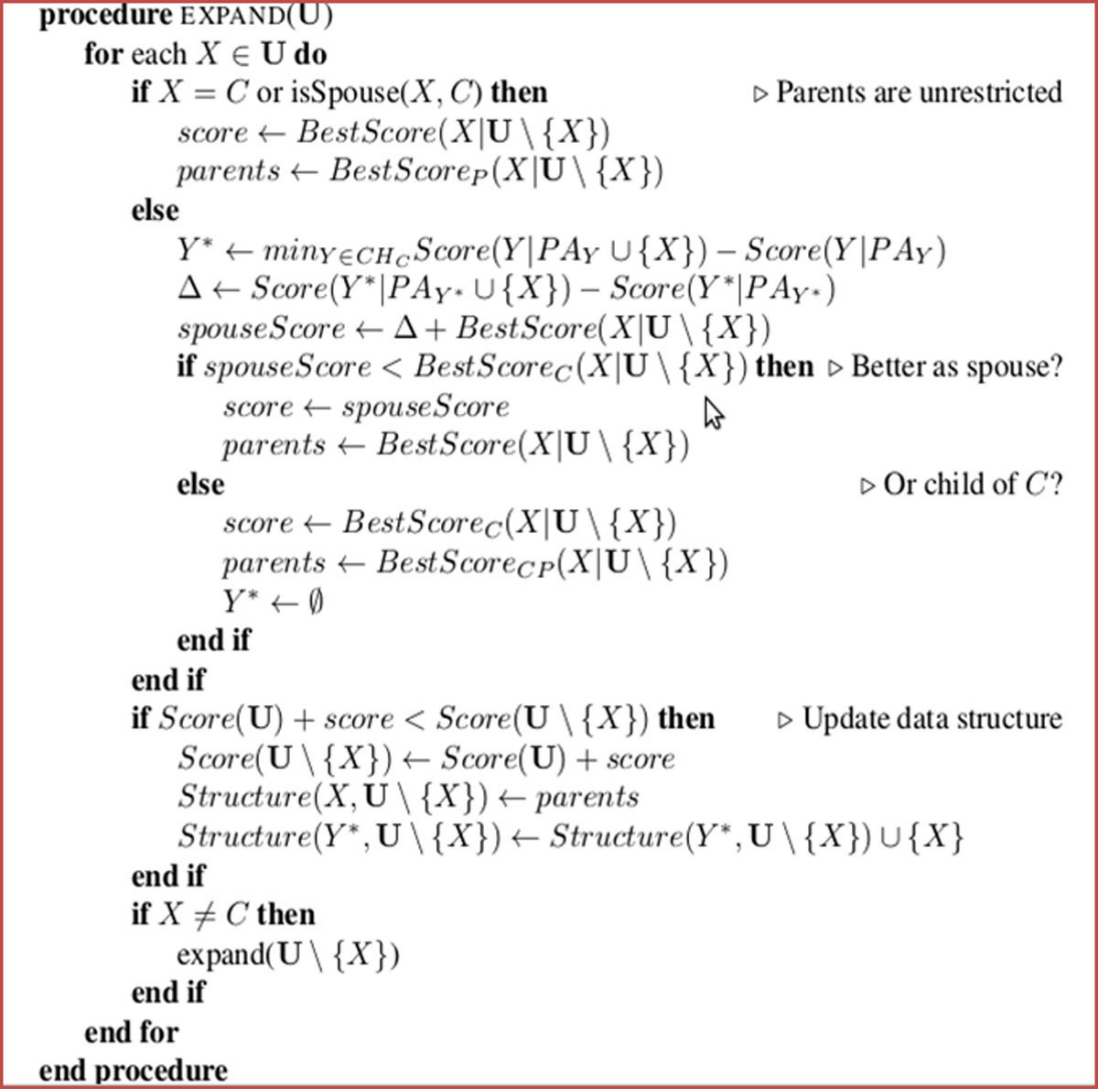
The implementation of Markov Blanket Feature Selection Algorithm.

The intuition of this algorithm is that an attribute is either a parent, child or spouse of *C*, or the attribute is not in *C*’s MB. Hence we only add each attribute to the MB according to an ordering and score for the new network. And we do not add attributes that make the score worse. Meanwhile, we keep the MB with the best score among all orderings. To this end, our algorithm performs feature selection and finds MB structure which has the maximum classification ability. The return structure is a Bayesian classifier for classification variable C. The general idea of the algorithm is shown in the Figure 1.

## Experiments

**Figure 2:**
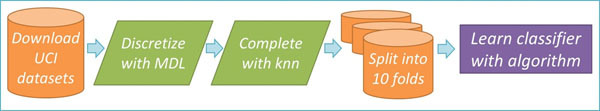
The procedure of data processing and classification.

We compared our feature selection algorithm to several state of the art classification methods on several benchmark datasets. All of the classification methods we selected learn a “human readable” model. In order to represent a wide variety of data domains, we downloaded 14 datasets from UCI machine learning repository [5]. The data processing and the classification steps in Figure 2 was followed a similar data procedure in [6].

## Results

**Figure 3:**
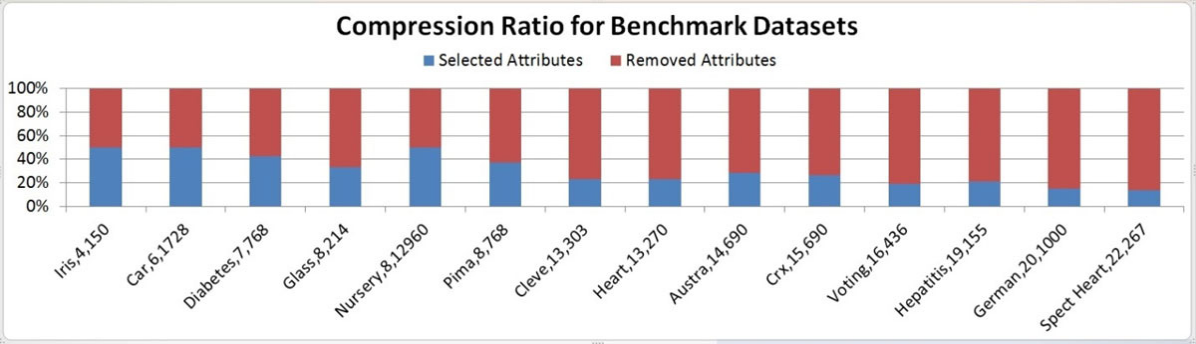
The Compression Ratio for Benchmark Datasets by Markov Blanket Feature Selection Algorithm.

As shown in Figure 3 for compression ratio of these benchmark datasets, our feature selection often achieved quite high compression ratios by ignoring attributes which do not help predict C. From this, we infer that only a few attributes are necessary to predict C.

**Figure 4:**
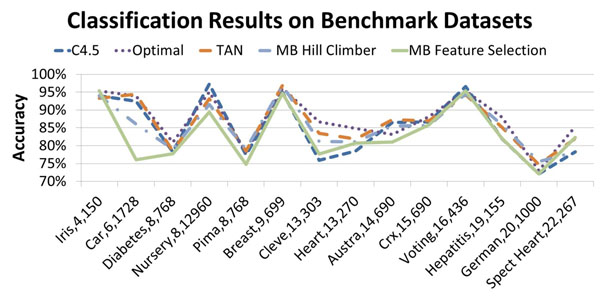
The classification results for Benchmark Datasets by Markov Blanket Feature Selection Algorithm.

The accuracy results in Figure 4 demonstrate that, despite compressing the data over 80% in some cases, MB feature selection is still competitive in terms of accuracy with state of the art methods.

## Discussion and conclusions

The compression ratio decreases as the number of variables in the dataset increases. This suggests that, even as dataset sizes increase, only a few attributes are helpful in predicting the class variable. The compression ratio is unaffected by the number of records in the dataset. This suggests that even when given many records, our algorithm does not pick many attributes in an attempt to overfit the dataset. Ignoring unimportant attributes does not significantly affect the classification accuracy. Despite compressing the data on average more than 70%, the classification accuracy is rarely more that 5% below the best classifier. Identifying MB variables could significantly reduce the cost of diagnostic lab tests by focusing interest on only the most relevant attributes.
